# The primary study of low-dose pancreas perfusion by 640- slice helical CT: a whole-organ perfusion

**DOI:** 10.1186/s40064-015-0950-6

**Published:** 2015-04-21

**Authors:** Zhengwu Tan, Qi Miao, Xiaoling Li, Ke Ren, Yu Zhao, Li Zhao, Xuedan Li, Yi Liu, Ruimei Chai, Ke Xu

**Affiliations:** Department of Radiology, The first affiliated hospital, China Medical University, Shenyang, Liaoning Zip code:110001 PR China; Department of Radiology, The first affiliated hospital, Heilongjiang University of Chinese Medicine, Haibin, Heilongjiang Zip code:150040 PR China

**Keywords:** Pancreas, Whole-organ, 640-slice helical CT, Perfusion, Low dose

## Abstract

To discuss the feasibility of low-dose whole-pancreas imaging utilizing 640-slice dynamic volume CT.80 patients (40 cases of normal pancreas and 40 patients supposed of having pancreatic carcinoma or focal pancreatic space-occupying lesions were mainly refered) referred for CT pancreas perfusion were enrolled in the study. 80 patients randomly assigned to 3 groups: Group ① (whole sequence). Group ② (odd number sequence). Group ③ (even number group)(Compared to ①, the scanning times and effective radiate dose of ② and ③ decreased about 50% respectively). The head, body, tail of each normal pancreas without any pancreatic disease, lesion and lesion-surrounding areas of each pancreatic cancer were selected as ROI, and tissue peak, blood flow are measured.According to pathology and clinical materials, 27 patients were diagnosed as pancreatic cancer; 40 patients were diagnosed as normal pancreas. The tissue peak and blood flow of the head, body, tail of normal pancreas without any pancreatic disease are 109.63 ± 16.60 and 131.90 ± 41.61, 104.38 ± 19.39 and 127.78 ± 42.52, 104.55 ± 15. 44 and 123.50 ± 33.44 respectively. The tissue peak and blood flow of pancreatic cancer is 59.59 ± 18.20 and 60.00 ± 15.36. For and between each group, there is no significant statistical difference for the tissue peak and blood flow of normal areas of the head, body, tail of normal pancreas. There is statistical difference for the tissue peak and blood flow of lesion and lesion-surrounding areas of pancreatic cancer in each group. However, there is no statistical difference for the tissue peak and blood flow of normal and diseasing areas between 3 groups.Low-dose whole-pancreas perfusion with 640-slice dynamic volume CT is feasible.

## Introduction

With the development of wide coverage CT, CT-perfusion us increasing popular, and applied to abdominal organs, including liver, kidney, pancreas (Pandharipande et al. 2005; Van Beers et al. 2001; Daghini et al. 2007; Ma et al. 2008). For technical reasons, such as the width-restrained detector, organ condition, and high-dose which is still the primary consideration, the application of CT-perfusion is clinically limited (Schindera et al. 2008; Nakayama et al. 2005). However, a 640-slice CT (Aquilion One: Toshiba Medical Systems) makes rapid scanning, low-dose and whole-organ perfusion possible. In this study, we evaluated the feasibility of low-dose scanning through comparing changes of tissue peak and blood flow of normal tissues, lesions and its surrounding areas of each pancreas.

## Subject and methods

### Subject

From March of 2013 to December of 2014, 40 patients chosen from outpatients and inpatients and clinically supposed to have pancreatic cancer or other pancreatic occupying disease were collected to undertake Toshiba640- slice dynamic volume CT for pancreatic perfusion. Half of them are males and half of them are females. Their age varies from 17 to 72 years old, and the average age is 54 years old. All patients received therapy in hospital post-scanning. They all had been proved to have no renal dysfunction and allergy to contrast material. All patients and their next of kin provided written informed medical consent to the study. The study was reviewed and approved by the ethics committee before the study began. Meanwhile, collected 40 cases of normal pancreas cases without any pancreatic disease to control group, 25 males and 15 females, Their age varies from 20 to 67 years old, and the average age is 45 years old.

### Scanning methods

For successful operation, we elaborated the perfusion indications, adverse reactions, operation procedures and gastrointestinal preparation methods to patients before scanning. The contrast agent (Iohexol 320 mg/ml) is injected via right medial cubital vein. Toshiba 640- slice dynamic volume CT is selected as the scanning apparatus. The scanning parameters: tube voltage 100 kV, tube current 50 mA, slice thickness 0.5 mm, scanning range 160 mm, pitch 87, matrix 512 × 512, volume scan image reconstruction with adaptive iterative dose reduction 3D (AIDR 3D) algorithm. For each patient,40 ml Iohexol and 40 ml physiological saline are injected with the flowing rate of5.0 ml/s. The contrast agent injection and scanning began at the same time to ensure acquisition of two groups of imaging (equivalently to plain scan ) before the enhancement of aorta. The whole perfusion took 36 s, composing of 12 sequences. 12 sequences: 2, 10, 12, 14, 16, 19, 21, 23, 26, 29, 31, 36 s.

### Imaging diagnosis

40 patients supposed to have pancreatic cancer or other pancreatic occupying disease performed pancreatic CT-perfusion. According to radiologic reports, there were 28 cases of pancreatic cancer, 2 cases of pancreatitis, 1 case of retroperitoneal lymphoma with tail pancreatic invasion, 1 case of solid-pseudopapillary tumor of pancreas, 2 cases of islet cell tumor, 1 case of duodenal cancer, and 5 normal cases.

### Image processing and analysis

Images were then transferred to a PC using the newly introduced enhanced CT DICOM protocol. Post-processing started on our inhouse developed software(H.M.) with images registration as the first step, focusing on the pancreas in order to correct for motion and breathing differences in all three planes. Each patient had 12 raw image sequences, and we defined sequences 1–12 as Group ① (whole sequence); sequences composed of 1st, 3rd, 5th, 7th, 9th, and 11th as Group ② (odd number sequence); and sequences composed of 2nd, 4th, 6th, 8th, 10th, and 12th as Group ③ (even number sequence). Then, we analyzed all sequences by maximum slope method (Sorensen et al. 1999). Abdominal aorta, normal areas of the without any pancreatic disease head, body, tail and lesion, lesion-surrounding areas of with pancreatic disease were selected as ROI, and the time-density curve of each ROI was calculated. ROI should be placed in the middle of normal pancreas or in strengthening area with lesion, avoiding the edging area as far as possible or the necrosis area lesion. The range of each ROI was about 1 ~ 3 mm^2^. The time-intensity curve of abdominal aorta, normal pancreatic areas was taken as reference standard to generate pseudo color, and then the responding blood flow is calculated. Herein, head pancreatic was generally chosen to substitute normal pancreatic areas. Once space-occupying lesion was found in head pancreatic, the head pancreatic adjacent areas or body pancreatic was selected.

### Radiation dose

For each patient, the total radiation dose one received was defined as Effective Radiation dose (mSv). Radiation dose = DLP · *k*, herein, DLP (mGy•cm) was dose-length. For abdominal scan, *k* was about 0.015 (CEC 1999). The post-processing and reading of images were finished by veteran technicians and chief physicians from department of radiology.

### Statistical analysis

Data were statistically analyzed by SPSS13.0. Quantitative Data were expressed as mean ± S.D. Differences were considered significant at a level of α = 0.05.

## Results

**Figure 1:**
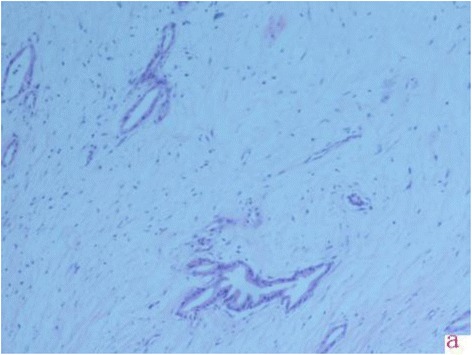
Pathology results. a represents the pathology results images of pancreatic cancer.

40 patients by surgical pathologically proven and clinical diagnosis. There were 27 pancreatic cancer patients, of which, 16 cases ( 10 cases located in head, and 6 cases in body of pancreas ) were pathologically proven (Figure 1), 11cases (7 cases located in head, 3 cases in body and 1 case in tail of pancreas ) proven by follow-up and clinical behaviors, and 5 cases accompanying with liver metastasis. 1 case of solid-pseudopapillary tumor, 1 case of duodenal cancer and 2 cases of islet cell tumor were pathologically proven. The remaining 4 cases did not perform surgery, and were diagnosed as cystoadenoma, cystic tumor, nonfunctional islet cell tumor or solid-pseudopapillary tumor according to clinical behaviors.

**Figure 2:**
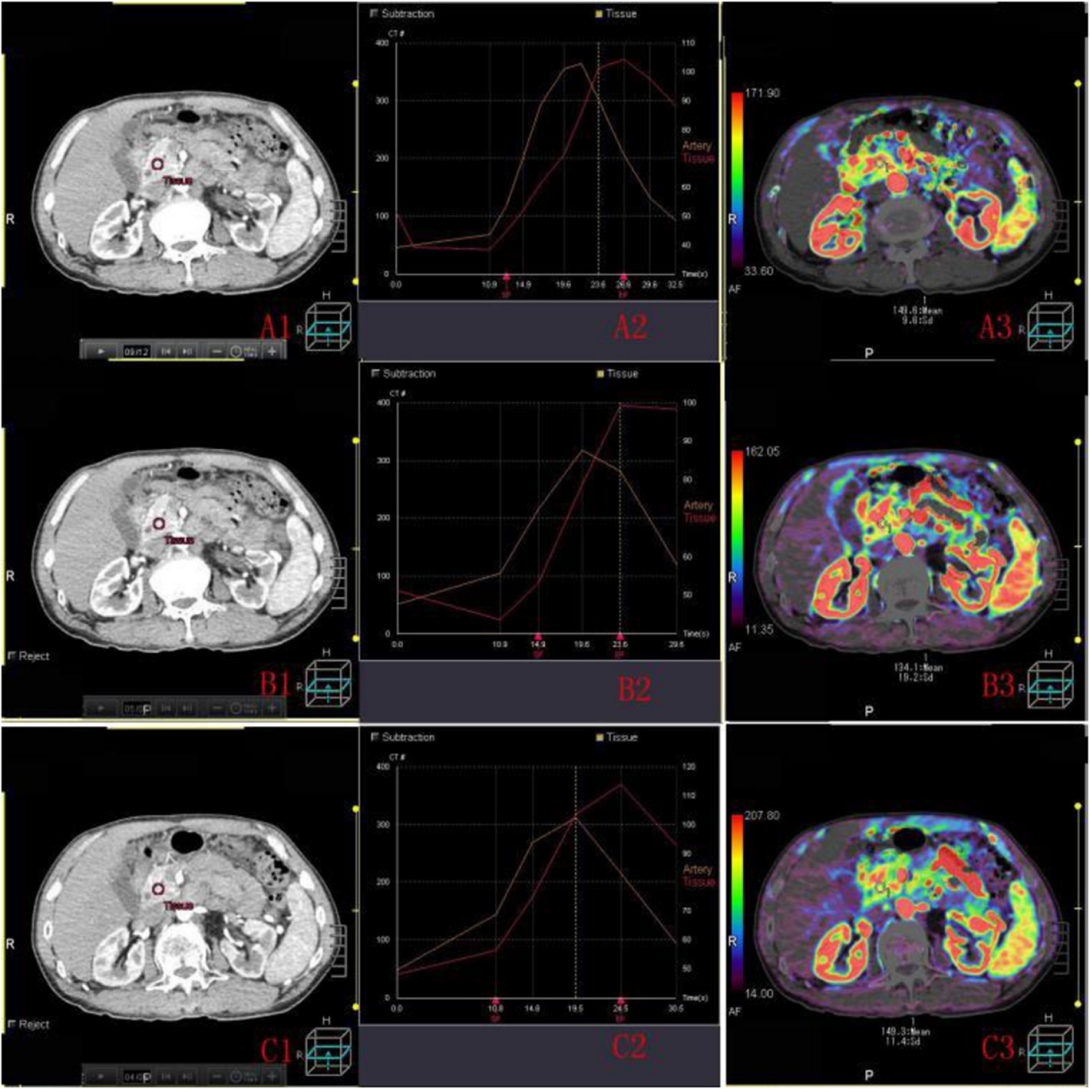
ROI, time density curve and blood flow image of normal pancreas. **A1(B1,C1)** shows the ROI of normal pancreas without any pancreatic disease in whole sequence (odd number sequence,even number group). **A2 (B2,C2)** represents the corresponding time density curve; **A3 (B3,C3)** represents the blood flow image.

**Figure 3:**
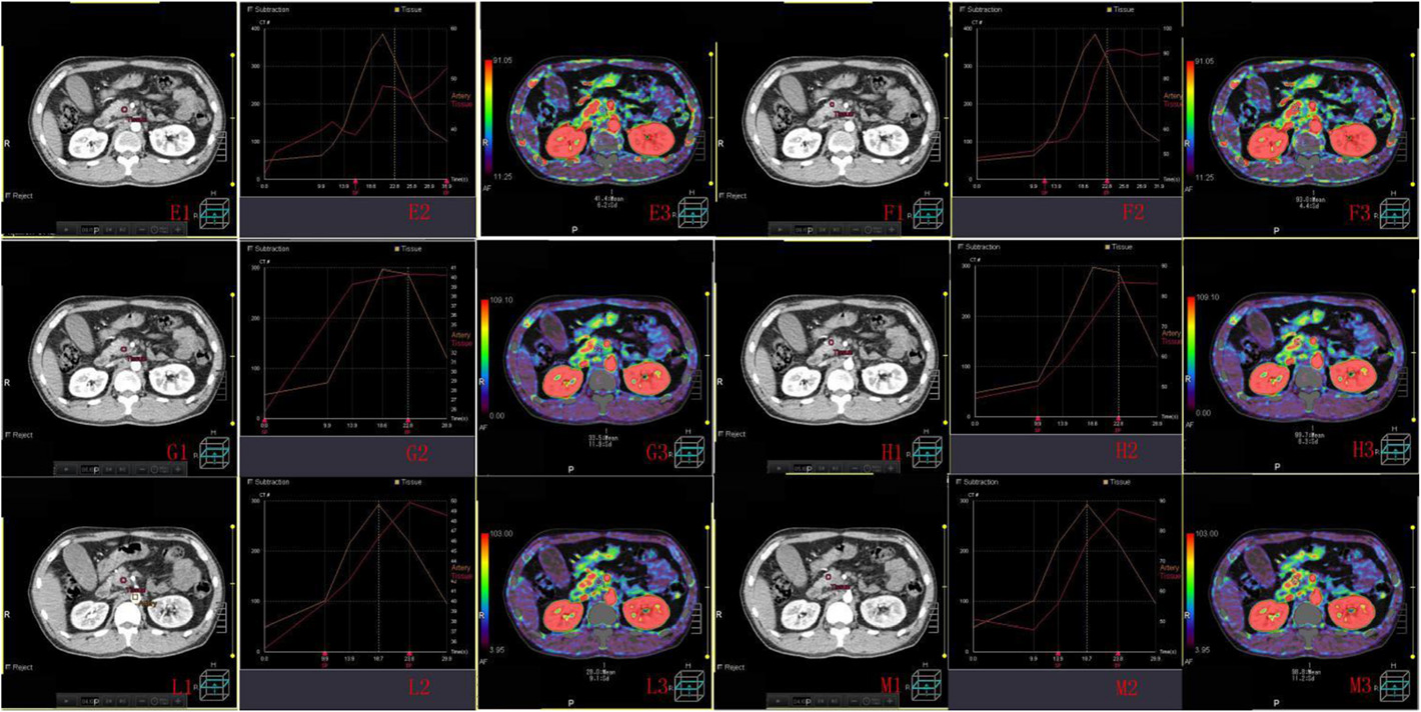
ROI, time density curve and blood flow image of pancreatic cancer (and the area adjacent to pancreatic cancer). **E1, G1, L1 (F1, H1, M1)** shows the ROI of pancreatic cancer (and the area adjacent to pancreatic cancer) in whole sequence,odd number sequence,even number group. **E2, G2, L2 (F2, H2, M2)** represents the corresponding time density curve; **E3, G3, L3 (F3, H3, M3)** represents the blood flow image.

Compare the tissue peak, blood flow of the head, body, tail areas of normal pancreas without any pancreatic disease, lesion and lesion-surrounding areas of pancreatic cancer in 3 groups and inner 3 groups (Figures 2 and 3 and Tables 1, 2, 3 and 4).

**Table 1:** **The statistical analysis of tissue peak, blood floe of norma areas of 40 cases of pancreatic without any pancreatic disease head, body and tail in 3 groups**

**ROI**	**Perfusion parameter** **(** $\overline{\mathbf{x}}\pm \mathbf{s}$ **)**	**Group** ①	**Group** ②	**Group** ③	**F value**	**P value**
Head pancreatic	Tissue peak	109.63 ± 16.60	104.58 ± 14.58	103.45 ± 14.60	1.850	0.162
	BF	131.90 ± 41.61	127.48 ± 68.54	139.98 ± 86.56	0.346	0.708
Body pancreatic	Tissue peak	104.38 ± 19.39	100.33 ± 17.96	98.38 ± 16.33	1.165	0.316
	BF	127.78 ± 42.52	121.48 ± 67.38	130.48 ± 76.98	0.209	0.812
Tail pancreatic	Tissue peak	104.55 ± 15.44	101.43 ± 17.17	100.08 ± 14.35	0.855	0.428
	BF	123.50 ± 33.44	121.48 ± 77.77	131.88 ± 88.32	0.244	0.784

**Table 2:** **The statistical analysis of tissue peak, blood flow of normal areas of 40 cases of pancreatic without any pancreatic disease head, body and tail inner 3 groups**

**Group**	**Perfusion parameter** **(** $\overline{\mathbf{x}}\pm \mathbf{s}$ **)**	**Head pancreatic**	**Body pancreatic**	**Tail pancreatic**	**F value**	**P value**
Group ①	Tissue peak	109.63 ± 16.60	104.38 ± 19.39	104.55 ± 15.44	1.199	0.305
	BF	131.90 ± 41.61	127.78 ± 42.52	123.50 ± 33.44	0.455	0.636
Group ②	Tissue peak	104.58 ± 14.58	100.33 ± 17.96	101.43 ± 17.17	0.703	0.497
	BF	127.48 ± 68.54	121.48 ± 67.38	121.48 ± 77.77	0.094	0.910
Group ③	Tissue peak	103.45 ± 14.60	98.38 ± 16.33	100.08 ± 14.35	1.167	0.315
	BF	139.98 ± 86.56	130.48 ± 76.98	131.88 ± 88.32	0.149	0.862

**Table 3:** **The statistical analysis of tissue peak, blood flow of 27 cases of pancreatic cancer and cancer-surrounding areas in 3 groups**

**ROI**	**Perfusion parameter** **(** $\overline{\mathbf{x}}\pm \mathbf{s}$ **)**	**Group** ①	**Group** ②	**Group** ③	**F value**	**P value**
Pancreatic cancer	Tissue peak	59.59 ± 18.20	61.93 ± 19.73	59.04 ± 18.49	0.179	0.836
	BF	60.00 ± 15.36	56.22 ± 20.56	60.81 ± 26.85	0.353	0.704
Cancer-surrounding areas	Tissue peak	101.11 ± 12.19	98.33 ± 12.35	99.00 ± 12.71	0.368	0.693
	BF	108.19 ± 21.77	103.67 ± 21.75	113.07 ± 30.08	0.968	0.384

**Table 4:** **The statistical analysis of tissue peak, blood flow of 27 cases of pancreatic cancer and cancer-surrounding areas inner 3 groups**

**Groups**	**Perfusion parameter** **(** $\overline{\mathbf{x}}\pm \mathbf{s}$ **)**	**Pancreatic cancer**	**Cancer-surrounding areas**	**T value**	**p value**
Group ①	Tissue peak	59.59 ± 18.20	101.11 ± 12.19	−11.141	0.000
	BF	60.00 ± 15.36	108.19 ± 21.77	−9.992	0.000
Group ②	Tissue peak	61.93 ± 19.73	98.33 ± 12.35	−9.731	0.000
	BF	56.22 ± 20.56	103.67 ± 21.75	−12.084	0.000
Group ③	Tissue peak	59.04 ± 18.49	99.00 ± 12.71	−10.586	0.000
	BF	60.81 ± 26.85	113.07 ± 30.08	−12.259	0.000

In our study, each patient got 12 scanning sequences, i.e. 12 data packages. Table 1 shows that the tissue peak and blood flow of head, body, tail areas of normal pancreas without any pancreatic disease are 109.63 ± 16.60 and 131.90 ± 41.61, 104.38 ± 19.39 and 127.78 ± 42.52, 104.55 ± 15. 44 and 123.50 ± 33.44 in Group ①, and there is no statistic significance with the values from Group ②, Group ③. Table 2 compares the tissue peak, blood flow of the head, body, tail areas of normal pancreas without any pancreatic disease from Group ①, Group ②, Group ③,There is no statistic significance between the three areas no matter which scan aequence we apply. Table 3 shows the tissue peak and blood flow of lesion and lesion-surrounding areas of pancreatic cancer is 59.59 ± 18.20 and 60.00 ± 15.36, 101.11 ± 12.19 and 108.19 ± 21.77 in Group ①, and there is no statistic significance with the values from Group ②, Group ③. Table 4 delineates there are statistical differences of tissue peak, blood flow between lesion and lesion-surrounding areas of pancreatic cancer in Group ①, Group ②, Group ③, the tissue peak and blood flow of lesion areas of pancreatic cancer is significantly lower than lesion-surrounding areas.

## Discussion

In 1991, Miles who was one of the peers to define the concept of CT-perfusion, undertook abdominal organ’s CT perfusion, and established artificial picture (Miles et al. 1991). In 1995, Miles initiated the application of CT perfusion method in pancreatic diseases and measured the perfusion parameters (blood flow, blood volume, time to peak, permeability picture, and Patlak blood volume) of normal pancreatic tissue, different lesions (Miles et al. 1995). In recent years, CT perfusion was clinically valuable in diagnosis, differential diagnosis of pancreatic tumors and the prognosis of transplanted pancreas. However, the high radiation dose of multi-slice CT has restrained its practical application in the past (Valentin 2007; HaU 2002; Hart et al. 2009; ICRP 2008; Li et al. 2014). With advancing of CT imaging technology, application of CT perfusion in pancreatic tumors is increasing popular. Our research aims at the feasibility study of low-dose whole-pancreas CT perfusion.

Each patient got 12 scanning sequences, i.e. 12 data packages. From Table 2, we conclude that different normal pancreatic areas, there is no discrimination in its tissue peak and blood flow. The data from Table 4 suggests the pancreatic cancer^,^s tissue peak and blood flow is significantly different from its lesion-surrounding areas of pancreatic cancer, and the former is lower than the later. Pancreas is an organ with rich blood supply, and its arterial blood supply comes from superior pancreaticoduodenal artery, inferior pancreaticoduodenal artery, dorsal pancreatic artery, transverse pancreatic artery and branches of splenic artery. The blood supply of head pancreatic mainly comes from superior and inferior pancreaticoduodenal artery, and their branches converge into anterior, posterior pancreaticoduodenal arcade. the splenic artery mainly supplies the body and tail of the pancreas (Takeda et al. 2005). Although there is discrimination of arterial blood supply, the difference of blood flow and tissue peak of different pancreatic areas is not obvious (Xu et al. 2009; Xie et al. 2013). As shown in Tables 2 and 4 , our results are in agreement with most previous reports (Xu et al. 2009. However, someone (Tsushina and Kusano 1998) reports the arterial blood supply in head pancreatic is lower than that of tail pancreatic. It is known to all that pancreatic cancer is in shortage of arterial blood supply. As shown in Table 4, the tissue peak and blood flow of pancreatic cancer areas is 59.59 ± 18.20 and 60.00 ± 15.36, lower than cancer-surrounding areas, and it’s in accordance with that pancreatic cancer has low blood supply. The lower tissue peak and blood flow results from the decreased permeability, low blood flow of pancreatic cancer, and increased interstitial tissues in tumor (Ma et al. 2008; McNulty et al. 2001; Miles et al. 2000; Ikeda et al. 1999). One defect of CT perfusion is its high radiation dose. Therefore, we propose to reduce tube current and scanning sequences (Ma et al. 2008; Xu et al. 2009; Xie et al. 2013; Tsushina and Kusano 1998; McNulty et al. 2001; Miles et al. 2000; Ikeda et al. 1999; Hashimoto et al. 2006). The CT images qualification was diagnostic satisfying with tube current being 50 mA. In previous reports (Xu et al. 2009; Xie et al. 2013 Tsushina and Kusano 1998; McNulty et al. 2001; Miles et al. 2000; Ikeda et al. 1999; Hashimoto et al. 2006), different tube currents were also applied, but they were most higher than 100 mA. However, Kadel S et al. (2009). used 45 mA tube current (100-kV tube voltage) on 320-slice helical CT and the total radiation exposure was 14.7 mSv. When only odd number sequence or even number sequence was performed, the radiation dose becomes half of that when Group ① was performed. Tables 1 and 3 shows that, the effective radiation dose for Group ① method is 13.51 ± 0.52 mSv, Group ② or Group ③ is 6.75 ± 0.27 mSv. In the each group, there is no difference in tissue peak and blood flow, though scan sequences, radiation dose decreased in group ②,③ compared with group ①. This shows that scanning method ② and ③ have no influence on the final results; thus, semi-dose ( low-dose ) scanning method is acceptable.

The narrow detector, respiratory motion from patients, long scanning time limited the application of CT perfusion in the past. We adapt dynamic volume scan mode. 320 images of 0.5 mm-thick slices are acquired after a single axial scan with a range of 160 mm. The large scan range covers not only pancreas but also the organs adjacent. Therefore, Toshiba 640- slice dynamic volume CT can shorten the scanning time and decrease the radiation dose. The CT perfusion analysis is not based on the high quality images from each volume data but on the intensity disparities emerging from different image groups. So the rapid and halve-dose scanning method does not impair image diagnosis (Kandel et al. 2009).

At present, the diagnosis of pancreatic cancer mainly depends on qualitative results from the under enhanced lesions observed in contrast enhanced CT scanning. However, a majority of pancreatic diseases are insufficient in blood supply, except for neuroendocrine tumors. Thus, for the lesions with insufficient blood supply, further quantitative diagnosis is needed. In light of the fact, a better diagnostic method is wanted. According to curve trend, tissue peak and blood flow, in descending order, we can not define a concrete critical value responding to different pancreatic diseases. One deficiency of this study is the small number of cases, which makes the CT perfusion’s differential diagnosis capacity ambiguously. Even though, the perfusion images acquired in this study composed of time-intensity curve, blood flow, render more information than previous pancreas perfusion (D’Onofrio et al. 2013).

## Conclusion

In conclusion, by using the method of low-dose whole-pancreas perfusion, scan sequences and radiation dose are halved, and the diagnosis capacity is not impaired. The curve slope, tissue peak, blood flow is approximately equivalent to previous reports. Thus, low-dose whole-pancreas perfusion is desirable to be an important method to diagnose pancreatic diseases, especially evaluating effects of early treatment for malignant tumors.
